# 567. The Quality & Management of Penicillin Allergy Labels in a Pediatric Birth Cohort

**DOI:** 10.1093/ofid/ofac492.620

**Published:** 2022-12-15

**Authors:** Margaret G Taylor, Torsten Joerger, Sara Anvari, Yun Li, Jeffrey S Gerber, Debra Palazzi

**Affiliations:** Baylor College of Medicine, Houston, Texas; Stanford University School of Medicine, Stanford, California; Baylor College of Medicine / Texas Children's Hospital, Houston, Texas; University of Pennsylvania, Philadelphia, Pennsylvania; Children's Hospital of Philadelphia, Philadelphia, Pennsylvania; Baylor College of Medicine, Houston, Texas

## Abstract

**Background:**

Penicillin allergy labels (PALs) influence antibiotic prescribing, yet little is known about the quality and management of PALs placed in the pediatric outpatient setting.

**Methods:**

We performed a retrospective chart review of 500 randomly selected children with PALs from a ten-year birth cohort (n=18,015) from Texas Children’s Pediatrics and Children’s Hospital of Philadelphia Primary Care Networks. PALs were classified as “not allergy” (family history, isolated diarrhea), “low risk” (maculopapular rash >24 hours into antibiotic course, no hives or pruritis), “moderate or high risk” (hives or pruritis, maculopapular rash < 24 hours into antibiotic course, anaphylaxis, swelling, or respiratory symptoms), “severe cutaneous reaction” (erythema multiforme, serum sickness), or “unable to classify” (information not available or unspecified rash characteristics or timing) based on 1) information in the allergy tab and 2) encounter notes. We used kappa-statistic measure-of-agreement to determine allergy classification agreement between the allergy tab and notes.

**Results:**

The median age of PAL placement was 1.4 years (IQR 0.9, 2.2). Most (n=303, 63%) PALs were placed by physicians. Half of PALs were categorized as “unable to classify; physicians were just as likely to place ambiguous PALs as other providers (48 vs 53%, p=0.31). Most (80%) children were evaluated at a primary care office within 1 week of their reaction. There was fair agreement between the allergy tab and encounter note documentation (Table 1). Of 54 (11%) children evaluated by an allergist, 25/25 passed an oral amoxicillin challenge. Only 69 (14%) children were de-labeled during the study period, more by an allergist (48%) than in the community (9.6%, p< 0.001).

**Table 1.**
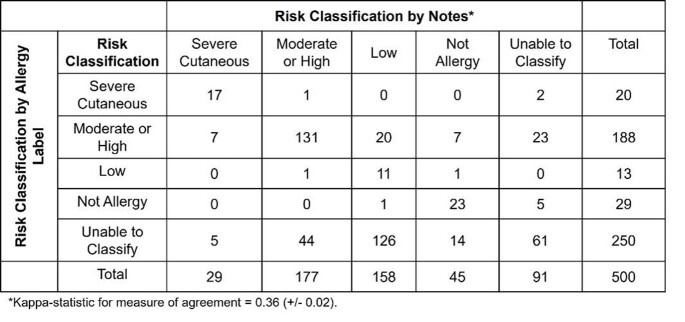
Risk stratification by penicillin allergy label and notes.

**Conclusion:**

PAL classification was ambiguous in half of labeled children; there was fair risk classification agreement between the allergy tab and notes. Children with PALs were rarely referred to allergists or de-labeled in the community. Future quality improvement efforts should seek to improve PAL documentation and access to allergy services.

**Disclosures:**

**Debra Palazzi, MD, MEd**, AAP: Board Member|AHRQ: Grant/Research Support|AMA: Board Member|Elsevier: Honoraria.

